# Supplementary material for: Automatic detection of adult cardiomyocyte for high throughput measurements of calcium and contractility
Source: PLoS One. 2021 Sep 1;16(9):e0256713. doi: 10.1371/journal.pone.0256713 (PMC8409674; doi:10.1371/journal.pone.0256713)
Supplement: S1 File — (PDF) [file pone.0256713.s001.pdf]

## S1 File: Static Cell Detection

```
/// <summary>
/// EdgeDetection cell finding algorithm.
/// </summary>
public class EdgeDetection
{
    /// <summary>
    /// Retrieve cells based on provided parameters from given image data.
    /// </summary>
    /// <returns>Constructed list of found cells in the image.</returns>
    public IList<CellMetric> GetCells(
            CellDetectParameters parameters,
            byte[]  imageData,
            int width,
            int height,
            out Bitmap bitmap)
    {
        bitmap = null;

        var image = new Image<Gray, byte>(width, height)
        {
            Bytes = imageData
        };

        // Take the gradiant in the x and y directions.
        Image<Gray, Single> img = image.Convert<Gray, Single>();
        Image<Gray, Single> gx = img.Sobel(1, 0, 3);
        Image<Gray, Single> gy = img.Sobel(0, 1, 3);
        // Get Magnitude of the gradient image (Gmag).
        Image<Gray, Single> gMagnitude = img.CopyBlank();
        Image<Gray, Single> gAngle = img.CopyBlank();
        CvInvoke.CartToPolar(gx, gy, gMagnitude, gAngle);

        gMagnitude = gMagnitude.SmoothGaussian(5);
        Image<Gray, byte> gMag2 = gMagnitude.Convert<Gray, byte>();

        gMag2 = ContrastStrecher.ContrastStreching(gMag2, 0.004f);
        Gray gray = new Gray(255);
        CvInvoke.Threshold(gMag2, gMag2, 50d, 255d, ThresholdType.Otsu | ThresholdTy
pe.Binary);

        gMag2 = gMag2.Dilate(5);
```

```
        gMag2 = gMag2.Erode(5);
        // Size filter.
        double maxArea =
            parameters.MaxArea / Math.Pow(parameters.PixelToAngstromRatio, 2.0) * Math
.Pow(10.0, 8.0);
        double minArea =
            parameters.MinArea / Math.Pow(parameters.PixelToAngstromRatio, 2.0) * Math.
Pow(10.0, 8.0);

        double ratioLow = parameters.MinWidthHeightRatio;
        double ratioHigh = parameters.MaxWidthHeightRatio;

        Mat element =
            CvInvoke.GetStructuringElement(ElementShape.Rectangle, new Size(3, 3), new P
oint(1, 1));
        CvInvoke.MorphologyEx(
            gMag2,
            gMag2,
            MorphOp.Open,
            element,
            new Point(1, 1),
            20,
            BorderType.Default,
            new MCvScalar(0));

        // Define the contours in the image.
        Image<Gray, byte> resultImage = ImageContours.ContoursDefinition(image, gMag2
, minArea);

        var tempImage = resultImage.Copy();

        var cells = new List<CellMetric>();

        using (VectorOfVectorOfPoint contours = new VectorOfVectorOfPoint())
        {
            CvInvoke.FindContours(
                resultImage,
                contours,
                null,
                RetrType.Tree,
                ChainApproxMethod.ChainApproxSimple);
            for (int i = 0; i < contours.Size; i++)
            {
```

```
double contourArea = CvInvoke.ContourArea(contours[i]);

// Note: Minimal bounding box
RotatedRect rect = CvInvoke.MinAreaRect(contours[i]);

if (contourArea > maxArea || contourArea < minArea)
{
    // Area rejection criteria
    continue;
}

double w = rect.Size.Width;
double h = rect.Size.Height;
double ratio;
if (w > h)
{
    ratio = h / w;
}
else
{
    ratio = w / h;
}

if (ratio < ratioLow || ratio > ratioHigh)
{
    //Width/Height ratio rejection criteria
    continue;
}

// Check if the contour includes more than one cells.
var areaRatio = contourArea / (w * h);
if (areaRatio < 0.55)
{
    //Rejection based on region of interest rejecting two or more cells
    continue;
}

// Check if the contour is on the border.
PointF[] rectVertices = rect.GetVertices();
bool flag = false;
for (int k = 0; k < 4; k++)
{
    if (rectVertices[k].X <= 10 || rectVertices[k].Y <= 10 ||
```

```
    rectVertices[k].X >= img.Width - 10 ||
    rectVertices[k].Y >= img.Height - 10)
    {
        flag = true;
    }
}

if (flag)
{
    //Rejected cell which is on the border of the field of view
    continue;
}

double widthTmp;
double heightTmp;
double angleTmp;
if (rect.Size.Width < rect.Size.Height)
{
    widthTmp = rect.Size.Height;
    heightTmp = rect.Size.Width;
    angleTmp = rect.Angle + 90;
}
else
{
    widthTmp = rect.Size.Width;
    heightTmp = rect.Size.Height;
    angleTmp = rect.Angle;
}

var cell = new CellMetric
{
    X = rect.Center.X,
    Y = rect.Center.Y,
    Width = widthTmp,
    Height = heightTmp,
    Angle = angleTmp,
    Vertices = rect.GetVertices(),
    Center = rect.Center,

    SubRectHeight = 30,
    SubRectWidth = 100,
    SubRectX = rect.Center.X,
    SubRectY = rect.Center.Y,
```

```
                SubRectAngle = angleTmp
            };

            cells.Add(cell);
        }
    }

    return cells;
  }
}
```
