# Supplementary material for: Automatic detection of adult cardiomyocyte for high throughput measurements of calcium and contractility
Source: PLoS One. 2021 Sep 1;16(9):e0256713. doi: 10.1371/journal.pone.0256713 (PMC8409674; doi:10.1371/journal.pone.0256713)
Supplement: S2 File — (PDF) [file pone.0256713.s002.pdf]

S2 File: Motion Cell Detection

```
public class MotionFrame
{
   private IPluginEnvironment _environment;
   private AcquisitionGenericConfiguration _genericConfiguration;
   private MotionFrameConfiguration _configuration;
   private readonly AutoResetEvent _waitHandle = new AutoResetEvent(false);
   private IImageData _lastImage;
   private readonly object _lastImageLock = new object();
   private IImageData _srcImage;

   public void Initialize(
            IPluginEnvironment pluginEnvironment,
            AcquisitionGenericConfiguration genericConfiguration,
            object configuration)
   {
      _environment = pluginEnvironment;
      _genericConfiguration = genericConfiguration;
      _configuration = configuration as MotionFrameConfiguration;
   }

   public List<CellInfo> Detect()
   {
      var configuration = _environment.Configuration;
      var pixelToAngstromRatio = configuration.PixelToAngstromFactor;
      var systemController = _environment.SystemController;
      var frameRate = 20;
      var motionArray = FindMotionStdOnLive(frameRate / _configuration.StimulationFrequency
);

      var cellDetectParameters = new CellDetectParameters
      {
         MinWidthHeightRatio = _genericConfiguration.MinWidthHeightRatio,
         MaxWidthHeightRatio = _genericConfiguration.MaxWidthHeightRatio,
         MinArea = _genericConfiguration.MinArea,
         MaxArea = _genericConfiguration.MaxArea,
         PixelToAngstromRatio = pixelToAngstromRatio,
         OrgImg = _srcImage.Data
      };
      var analyser = DetermineAnalyser();
      var analysisResult = analyser.GetCells(
           cellDetectParameters,
           motionArray,
           _srcImage.Width,
           _srcImage.Height,
```

```
            out Bitmap bitmap);

        var posZ = systemController.GetPositionZ();
        var posXY = systemController.GetPositionXY();

        var result = new List<CellInfo>();
        foreach (var cell in analysisResult)
        {
            var x = Convert.ToInt32(posXY.X + (_srcImage.Width / 2 - cell.X) * pixelToAngstromRatio)
;
            var y = Convert.ToInt32(posXY.Y + (_srcImage.Height / 2 - cell.Y) * pixelToAngstromRatio
);
            var pos = new PositionXY(x, y);
            var angle = cell.Angle;
            result.Add(new CellInfo
            {
                Position = new PositionXYZ(pos, posZ),
                Angle = angle,
                ValidationPosition = new PositionXY(Convert.ToInt32(cell.X), Convert.ToInt32(cell.Y))
            });
        }

        return result;
    }

    private ICellDetectAlgorithm DetermineAnalyser()
    {
        ICellDetectAlgorithm analyser;
        switch (_configuration.CellDetectAlgorithm)
        {
            case MotionFrameConfiguration.CellDetectAlgorithms.MotionEdgeDetection:
                analyser = new MotionEdgeDetection();
                break;
            case MotionFrameConfiguration.CellDetectAlgorithms.MotionEnFCMEdgeDetection:
                analyser = new MotionEnFCMEdgeDetection();
                break;
            default:
                throw new PluginException("Unknown CellDetectAlgorithm");
        }

        return analyser;
    }

    private byte[] FindMotionStdOnLive(int framerate)
    {
```

```
//calculate standard deviation on live stream. take the first frame as K in the
    //algorithm.
byte[] K = new byte[0];
int[] Ex = new int[0];
int[] Ex2 = new int[0];
for (int i = 0; i < framerate; i++)
{
  var image = WaitImage();
  if (image != null)
  {
    if (i == 0)
    {
      int length = image.Data.Length;
      K = image.Data;
      Ex = new int[length];
      Ex2 = new int[length];
      _srcImage = image;
    }

    byte[] x = image.Data;
    for (int j = 0; j < K.Length; j++)
    {
      Ex[j] += x[j] - K[j];
      Ex2[j] += (x[j] - K[j]) * (x[j] - K[j]);
    }
  }
}

int[] variance = new int[K.Length];
for (int j = 0; j < K.Length; j++)
{
  variance[j] = (Ex2[j] - (Ex[j] * Ex[j]) / framerate) / (framerate - 1);
}
byte[] result = new byte[K.Length];
int minVar = variance.Min();
int maxVar = variance.Max();
if (minVar != maxVar)
{
  for (int j = 0; j < K.Length; j++)
  {
    int temp = 255 * (variance[j] - minVar) / (maxVar - minVar);
    result[j] = Convert.ToByte(temp);
  }
}
```

```
        return result;
    }
}
```
