# Supplementary material for: Automatic detection of adult cardiomyocyte for high throughput measurements of calcium and contractility
Source: PLoS One. 2021 Sep 1;16(9):e0256713. doi: 10.1371/journal.pone.0256713 (PMC8409674; doi:10.1371/journal.pone.0256713)
Supplement: S3 File — (PDF) [file pone.0256713.s003.pdf]

S3 File: Fine Auto Focus

```
public class FineAutoFocusFilter : IFilter
{
   private ISystemController _systemController;
   private IPluginEnvironment _environment;
   private FineAutoFocusFilterConfiguration _configuration;

   public void Initialize(
           IPluginEnvironment environment,
           AcquisitionGenericConfiguration genericConfiguration,
           object configuration)
   {
      _environment = environment;
      _configuration = configuration as FineAutoFocusFilterConfiguration;
      _systemController = _environment.SystemController;

   }

   public bool Adjust(CellInfo cellInfo)
   {
      var imageRotator = _environment.ImageRotationConnector;
      var cellManagement = _environment.CellManagement;

      // first move to the position and set the angle.
      _systemController.MoveAbsoluteXY(cellInfo.Position.XY);
      imageRotator.SetRotatedImageAngle(cellInfo.Angle);

      // Do a fine auto focus using OneDFFT maxFrequency.
      DoFineFocus();

      // Do a second round of auto focus using OneDFFT maxSNR.
      // Signal to noise ratio is only work for short range when it is already
      // really close to the optimal z-location.
      // It is not working for large range. That is also the reason why I have
      // two steps of fine autofocus.
      DoSecondFineFocus();

      PositionZ posZ = _systemController.GetPositionZ();
      PositionXY posXY = _systemController.GetPositionXY();
      cellInfo.Position = new PositionXYZ(posXY, posZ);

      return true;
   }
   private void DoFineFocus()
   {
```

```
            var currentZ = _systemController.GetPositionZ();
            DoubleSweepProcess autoFocus = new DoubleSweepProcess(
                  new ImageAnalyser(),
                  null,
                  FocusAlgorithm.OneDFFT,
                  currentZ.Z - _configuration.FirstAutoFocusRange*10000,
                  currentZ.Z + _configuration.FirstAutoFocusRange*10000,
                  _configuration.FirstAutoFocusSpeed);
            autoFocus.Execute();
        }

        private void DoSecondFineFocus()
        {
            var currentZ = _systemController.GetPositionZ();
            DoubleSweepProcess autoFocus = new DoubleSweepProcess(
                  new ImageAnalyser(),
                  null,
                  FocusAlgorithm.OneDFFTSNR,
                  currentZ.Z – _configuration.SecondAutoFocusRange*10000,
                  currentZ.Z + _configuration.SecondAutoFocusRange*10000,
                  _configuration.SecondAutoFocusSpeed);
            autoFocus.Execute();

        }

        public void CheckPredicates(IList<string> errors)
        {
            if (_environment.SarcomereControl == null)
            {
                errors.Add(
                  "The fine autofocus filter requires a sarcomere task to be present in IonWizard.");
            }
        }
    }
```
