# Supplementary material for: Automatic detection of adult cardiomyocyte for high throughput measurements of calcium and contractility
Source: PLoS One. 2021 Sep 1;16(9):e0256713. doi: 10.1371/journal.pone.0256713 (PMC8409674; doi:10.1371/journal.pone.0256713)
Supplement: S4 File — (PDF) [file pone.0256713.s004.pdf]

S4 File: Angle Correction

```
public class AngleCorrectionFilter
{
    private IPluginEnvironment _environment;
    private AngleCorrectionFilterConfiguration _configuration;
    private readonly AutoResetEvent _waitHandle = new AutoResetEvent(false);
    private IImageData _lastImage;
    private readonly object _lastImageLock = new object();
    private ISystemController _systemController;
    private double _pixelToAngstromRatio;

    public void Initialize(
            IPluginEnvironment pluginEnvironment,
            AcquisitionGenericConfiguration genericConfiguration,
            object configuration)
    {
        _environment = pluginEnvironment;
        _configuration = configuration as AngleCorrectionFilterConfiguration;
        _systemController = _environment.SystemController;
        _pixelToAngstromRatio = _environment.Configuration.PixelToAngstromFactor;
    }

    public bool Adjust(CellInfo cellInfo)
    {
        _waitHandle.Reset();

        var imageRotator = _environment.ImageRotationConnector;

        _systemController.MoveAbsoluteXY(cellInfo.Position.XY);
        imageRotator.SetRotatedImageAngle(cellInfo.Angle);

        try
        {
            IImageData imageData = GetNewImage();

            int rectWidth = 256;
            int rectHeight = 30;
            // Check the SNR before angle correction.
            // crop the image first to a rectangle of size regionSize.
              // Check the SNR before and after angle correction is because sometimes after
              // angle correction the SNR does not improve. Therefore, there is no need to
              // correct the angle

            Rectangle curRect = new Rectangle(
                    imageData.Width / 2 - rectWidth / 2,
```

```
                imageData.Height / 2 - rectHeight / 2,
                rectWidth,
                rectHeight);
        Bitmap imgBefore = CropRotatedRect.CropRotatedRectangle(
                imageData.GetAsBitmap(),
                curRect,
                (float)(-cellInfo.Angle),
                true);

        double snrBefore = SNRCalculation.ImgSNRCalculating(imgBefore, rectWidth, rectHeight)
;

        // Reorient the cells.
        var correctedAngle = Reorientation.GetAngleOffset(
                imageData,
                cellInfo.Angle,
                _pixelToAngstromRatio,
                rectWidth);

        float tmpCorrectedAngle = (float)(-correctedAngle);
        Bitmap imgAfter = CropRotatedRect.CropRotatedRectangle(
                imageData.GetAsBitmap(),
                curRect,
                tmpCorrectedAngle,
                true);
        double snrAfter = SNRCalculation.ImgSNRCalculating(imgAfter, rectWidth, rectHeight);

        // Reject dead cells.
        double snr = snrBefore > snrAfter ? snrBefore : snrAfter;
        if (snr < _configuration.SNRatio)
        {
            return false;
        }

        cellInfo.SignalToNoiseRatio = snr;

        if (snrAfter > snrBefore)
        {
            // Correct the Angle.
            imageRotator.SetRotatedImageAngle(correctedAngle);
        }
        else
        {
            correctedAngle = cellInfo.Angle;
        }
```

```
            // Update cellInfo angle.
            cellInfo.Angle = correctedAngle;
        }
        catch (Exception ex)
        {
            throw;
        }

        return true;
    }
}
```
