# Supplementary material for: Automatic detection of adult cardiomyocyte for high throughput measurements of calcium and contractility
Source: PLoS One. 2021 Sep 1;16(9):e0256713. doi: 10.1371/journal.pone.0256713 (PMC8409674; doi:10.1371/journal.pone.0256713)
Supplement: Attachment — Submitted filename: PLOS One automatic cell finding - Response to reviewers.docx [file pone.0256713.s005.docx]

Response to reviewers of the manuscript titled “Automatic detection of adult cardiomyocyte for high throughput measurements of calcium and contractility.”

*We thank the reviewers and the editor for their careful consideration of our work and appreciate the criticisms and recommendations, which we believe have helped to improve the manuscript.*

1. Please ensure that your manuscript meets PLOS ONE's style requirements, including those for file naming. The PLOS ONE style templates can be found at

<https://journals.plos.org/plosone/s/file?id=wjVg/PLOSOne_formatting_sample_main_body.pdf> and

<https://journals.plos.org/plosone/s/file?id=ba62/PLOSOne_formatting_sample_title_authors_affiliations.pdf>

*We have carefully checked the style requirements and have made some small modifications to the file naming and reference list.*

2. In the competing interests statement, please clarify why your affiliations to CytoCypher BV represent a competing interests.

*CytoCypher BV has commercialized the algorithm described in this manuscript producing cell finding software.*

3. Thank you for stating the following in the Competing Interests section:

"I have read the journal's policy and the authors of this manuscript have the following competing interests:L. Cao was an employee of CytoCypher BV, E. Manders is a part-time employee of CytoCypher BV and M.Helmes is the CEO of CytoCypher BV"

We note that one or more of the authors are employed by a commercial company: CytoCypher BV.

3.1. Please provide an amended Funding Statement declaring this commercial affiliation, as well as a statement regarding the Role of Funders in your study. If the funding organization did not play a role in the study design, data collection and analysis, decision to publish, or preparation of the manuscript and only provided financial support in the form of authors' salaries and/or research materials, please review your statements relating to the author contributions, and ensure you have specifically and accurately indicated the role(s) that these authors had in your study. You can update author roles in the Author Contributions section of the online submission form.

Please also include the following statement within your amended Funding Statement.

“The funder provided support in the form of salaries for authors [insert relevant initials], but did not have any additional role in the study design, data collection and analysis, decision to publish, or preparation of the manuscript. The specific roles of these authors are articulated in the ‘author contributions’ section.”

If your commercial affiliation did play a role in your study, please state and explain this role within your updated Funding Statement.

3.2. Please also provide an updated Competing Interests Statement declaring this commercial affiliation along with any other relevant declarations relating to employment, consultancy, patents, products in development, or marketed products, etc.

Within your Competing Interests Statement, please confirm that this commercial affiliation does not alter your adherence to all PLOS ONE policies on sharing data and materials by including the following statement: "This does not alter our adherence to PLOS ONE policies on sharing data and materials.” (as detailed online in our guide for authors http://journals.plos.org/plosone/s/competing-interests) . If this adherence statement is not accurate and there are restrictions on sharing of data and/or materials, please state these. Please note that we cannot proceed with consideration of your article until this information has been declared.

Please include both an updated Funding Statement and Competing Interests Statement in your cover letter. We will change the online submission form on your behalf.

Please know it is PLOS ONE policy for corresponding authors to declare, on behalf of all authors, all potential competing interests for the purposes of transparency. PLOS defines a competing interest as anything that interferes with, or could reasonably be perceived as interfering with, the full and objective presentation, peer review, editorial decision-making, or publication of research or non-research articles submitted to one of the journals. Competing interests can be financial or non-financial, professional, or personal. Competing interests can arise in relationship to an organization or another person. Please follow this link to our website for more details on competing interests: <http://journals.plos.org/plosone/s/competing-interests>

*We will add the Funding Statement to our submission. We have updated our Competing Interests Statement as suggested.*

Reviewer #1:

1) The model is demonstrated in a scenario with a low cell density (Fig. 3). I am wondering how the performance of the automatic segmentation masks would change in a scenario with high cell density. As the algorithm is based on edge detection, one might expect a drastic drop in segmentation performance (F1 score) in a scenario with overlapping cells / cell agglomerations where e.g. watershed seg. / deep learning models are needed.

*The algorithm is specifically designed for measurements of calcium and contractility of single cardiomyocytes. These measurements require the cells not to overlap or touch each other as that could influence these measurements. We have added the average cell density of the dishes used for validation of our method (see line 235). In the discussion we explain in more detail how differences in cell density could affect the precision and sensitivity of our algorithm. See lines 337-346.*

In Line 335 you are stating „Each frame that only contains overlapping cells or no viable cells is wasted time“.

Regarding that I have some questions:

How important are scenarios of high cell density (overlapping cells) for the measurements? Are only regions with low cell densities (no overlapping cells) of interest for the measurement? Is a measurement with a cell confluency of 100% / high cell density possible?

*Indeed, only regions with no overlapping cells are of interest for these measurements. These are isolated cardiomyocytes which are not kept in culture but are measured on day of isolation. It is possible to measure calcium and contractility in a confluent cell layer, for example from cardiomyocyte induced pluripotent stem cells. However, the method of finding regions of interest to measure in these constructs is different and should not be based on edge detection.*

Is there a post-processing step needed (manual or automated) to exclude data / frames containing these "unnecessary data" (overlapping cells / agglomerations / image artifacts)? In other words, what is the process for image data selection?

*The current cell finding algorithm described in this paper includes a filter for overlapping cells (see lines 172-176) and enables the user to set a threshold for the distance between cells.*

*We thank the reviewer for pointing this out. We have clarified this more in the revised manuscript on lines 135-137.*

2) In the Result section you mention that the total amount of data acquired were 190 videos. Out of these videos, 212 beating cells are obtained / measured (line 232 ff.). Do you have an estimation / do you know the number of total cells contained in these 190 videos? In other words, what is the rate of total cells to actually measured cells? Can you upload some sample videos / images?

*We thank the reviewer for pointing this out. We added the cell density of the dishes used (62, 58, 70, 73 = 65/mm2). Although, it should be noted that for these measurements we are only interested in non-overlapping contracting cells and the distribution can vary a lot within a dish; cells tend to cluster together in the middle of dish. In our opinion, the number of total cells within a dish is not that relevant to assess the performance of our algorithm. However, we do feel it is valuable information to the user to report the cell density see line 235. In addition, we added the videos for the validation to the data repository.*

Reviewer #2:

In this manuscript, Cao et al describe an image analysis workflow capable of automatically identifying viable isolated adult cardiomyocytes and extracting sarcomere shortening and Ca data from each identified cell. In conjunction with computer-controlled microscopy equipment, this image analysis approach enables a substantial increase in throughput (cells per unit time). It also removes experimental bias by using consistent and quantitative metrics for quality.

The work presented here is certainly of interest to the field and communicates some interesting and novel approaches to detecting and characterizing isolated cardiomyocytes. I have a few minor comments that I feel should be addressed:

*We thank the reviewer for his appreciation of our work.*

1. Lines 280-288: In describing data histograms, the authors speak of distributions being “shifted to the left” – this is a qualitative description that doesn’t mean anything to the reader. It would be much better to say something like “the distribution of fractional shortening measurements was shifted toward smaller values of shortening in the automatically found cells…”

*We agree with the reviewer that we could give a better description of the changes in the data histograms. We have adjusted this paragraph accordingly (Results, lines 285-286)*

2. Discussion, line 292: The authors mention that their approach “improved the data quality and resulted in more reliable hypothesis testing.” I would encourage the authors to be much more specific or to remove this language. It is not obvious what “quality” means, nor what hypotheses were being tested – I don’t see where the data prove that the automated testing resulted in a more reliable hypothesis test.

*We thank the reviewer for pointing this out, we agree that our wording was not specific enough. We have adjusted this paragraph accordingly (Discussion, lines 295-297)*

3. Discussion, lines 314-318: There are obviously various parameters in the image analysis that can be varied in order to optimize output. Are the values reported and used in this manuscript likely to work generally across projects, or will this have to be revisited for cells from different species, disease states, etc? It would be helpful to comment on this.

*You can set the various parameters in way they can be used across projects. The settings used in the manuscript functioned well for both mouse and rat isolations. For isolations from different species (i.e. human, porcine), you may require to revisit the settings. The settings can be saved and can easily be imported when starting an experiment to ensure the same settings are used within a project.*

4. Our own work has involved improving throughput of these isolated cardiomyocyte measurements. I noticed that exchange of solutions to alter drug presence was done manually by hand. We developed a microwell system for trapping cardiomyocytes in predictable orientations while also enabling continuous fluid flow (and exchange). Please see https://doi.org/10.1016/j.bpj.2019.08.024. It may be helpful to consider how this approach could be used to extend the methods presented here.

*Indeed, your work on trapping isolated cardiomyocytes could further improve the throughput of our algorithm. It ensures equal distribution of the cells throughout a dish and prevents or reduces the chances of cells overlapping. We address this in the discussion on lines 343-346.*
